# Interactive, Narrative-Based Digital Health Interventions for Vaccine Communication: Scoping Review

**DOI:** 10.3390/vaccines13121220

**Published:** 2025-12-02

**Authors:** Ahmed Haji Said, Fiona Syed, Isabelle Ma, Rida Akbar, Nidhi Ramprasad, Erin E. Reardon, Robert A. Bednarczyk, Kate Winskell, Lavanya Vasudevan

**Affiliations:** 1 Hubert Department of Global Health, Rollins School of Public Health, Emory University, Atlanta, GA 30322, USA; rida.akbardr@gmail.com (R.A.); robert.a.bednarczyk@emory.edu (R.A.B.); swinske@emory.edu (K.W.);; 2 Center for Medicare (CM), Centers for Medicare & Medicaid Services (CMS), U.S. Department of Health and Human Services (HHS), Baltimore, MD 21244, USA; 3 College of Arts and Sciences, Emory University, Atlanta, GA 30322, USA; isabelle.ma@emory.edu; 4 School of Medicine, Emory University, Atlanta, GA 30322, USA; 5 Woodruff Health Sciences Center Library, Emory University, Atlanta, GA 30322, USA; erin.reardon@emory.edu

**Keywords:** vaccines, vaccine communication, vaccine hesitancy, storytelling, digital health, interactivity

## Abstract

**Background:** Interactive, narrative-based digital health interventions may positively influence vaccination-related attitudes, intentions, and uptake. However, evidence on their implementation and evaluation for vaccine communication has not yet been comprehensively synthesized. Our research questions (RQs) were to describe the use of interactive, narrative-based digital health interventions for vaccine communication (RQ1), their impact on individuals’ vaccine intention or uptake (RQ2), and factors associated with their implementation (RQ3). **Methods:** A scoping review was conducted using Arksey and O’Malley’s 5-stage framework and the PRISMA-ScR guidelines. We searched PubMed, Embase, Scopus, Web of Science, CINAHL, and PsycINFO from inception to 18 April 2023. To be included in the review, studies had to include empirical findings from primary data collection, address vaccine communication, use narrative communication that enabled audience engagement (i.e., interactivity), and deliver the narrative through a digital health device or modality. **Results:** The search identified 6834 records, with 25 studies meeting the inclusion criteria. For RQ1, the interventions most often focused on HPV vaccination (12 studies). Communication objectives included addressing vaccine hesitancy and increasing vaccination confidence or knowledge. Intervention delivery formats included multi-device compatibility (utilizing more than one device type, 7 studies) and incorporated interactive features, such as gamification and push notifications. Invented narratives were the most frequent narrative approach (8 studies). For RQ2, vaccination outcomes were reported in nearly half the studies (12 studies), with vaccination intention assessed in 8 studies and both vaccination intention and uptake assessed in 4 studies. For RQ3, implementation factors were reported in nearly half the studies (12 studies), with the most frequently reported outcome being acceptability (6 studies). **Conclusions:** Evidence supporting interactive, narrative-based digital health interventions for vaccine communication can be strengthened by diversifying narrative strategies, expanding the range of interactivity modalities tested, and focusing on a broader range of vaccines. Further research is needed to assess the effectiveness of these interventions, particularly of vaccine uptake. The insights from this scoping review may inform the development of novel future interventions for vaccine communication. The generalizability of these findings may be limited by the small number of studies in some categories and the preponderance of studies from high-income settings.

## 1. Introduction

Vaccination is one of the most effective public health measures for preventing infectious diseases and reducing associated morbidity and mortality. Since the launch of the Expanded Program on Immunization in 1974, global vaccination initiatives are estimated to have saved 154 million lives, including 146 million children under the age of five [1]. However, the uptake of vaccines is influenced by a complex interaction between trust in vaccines, healthcare providers, and policymakers [2], exposure to misinformation, and socio-cultural dynamics, particularly in the context of (re)emerging infectious diseases and novel vaccine technologies [3]. These and related factors result in variations in vaccination decision-making, including selective vaccine refusal, delayed vaccine acceptance, or vaccine acceptance with underlying uncertainty [4]. Cultivating and preserving trust in vaccines and vaccination programs requires proactive and transparent communication that addresses knowledge gaps, anticipates emerging concerns, and counters misinformation.

Traditional public health and medical communication often rely on statistical evidence to convey information and persuade audiences. However, data-driven messages may not always resonate with individuals’ subjective experiences or cognitive processes [5]. Empirical research has demonstrated that narratives are often more persuasive than statistical messages in promoting health-related behavior change, encouraging engagement with public health interventions, and supporting the adoption of preventive measures [5,6,7]. Narratives that harness the power of storytelling are increasingly recognized as a viable strategy for building vaccine confidence, addressing misinformation, and fostering vaccine acceptance [8,9,10].

Hinyard and Kreuter define narratives [11] as “any cohesive and coherent story with an identifiable beginning, middle, and end that provides information about scene, characters, and conflict; raises unanswered questions or unresolved conflict; and provides resolution.” Narratives that include interactivity differ from passive storytelling by encouraging active engagement, which has been shown to increase transportation, identification, and immersion [12]. For this scoping review, interactive narratives were defined as “the active engagement of individuals with the narrative via digital health, aiming to raise awareness, empower behavior change, and ultimately lead to improved vaccination outcomes” [13]. Digital health refers to “the use of information and communications technology in support of health and health-related fields” [14]. Applied to vaccination programs, interactive, narrative-based digital health interventions may allow individuals to explore hypothetical scenarios, weigh the potential risks and benefits of their vaccination-related decisions, and reflect on the relevance of those decisions to real-world health outcomes.

Although growing interest in narrative-based approaches has led to a proliferation of digital tools aimed at improving vaccine communication [15], the evidence base remains fragmented. Existing studies vary widely in their target populations, design features, and outcome measures, and a comprehensive synthesis of the scope and implementation of interactive, narrative-based digital health interventions for vaccine communication is lacking. To address this gap, we conducted a scoping review to identify and describe the use of interactive, narrative-based digital health interventions for vaccine communication, their impact on vaccination intention or uptake, and factors associated with their implementation.

## 2. Materials and Methods

### 2.1. Overview

We conducted a scoping review and followed Arksey and O’Malley’s 5-stage framework [16]. Our findings are reported in accordance with the PRISMA extension for scoping reviews (PRISMA-ScR) [17]. The protocol for this scoping review has been peer-reviewed and published [13], with key details summarized below.

#### 2.1.1. Stage 1: Identifying Research Questions

The scoping review was guided by three research questions (RQs):

RQ1. How have interactive, narrative-based digital health interventions been used for vaccine communication?

RQ2. How have interactive, narrative-based digital health interventions been evaluated for promoting vaccination intention or uptake?

RQ3. What implementation factors are associated with the use of interactive, narrative-based digital health interventions for vaccine communication?

#### 2.1.2. Stage 2: Identifying Relevant Studies

A detailed search string was developed with assistance from public health informationists and included keywords related to narratives, digital health, and vaccine communication (Supplementary File S1). The search string was implemented in the following databases: PubMed, Embase, Scopus, Web of Science, CINAHL, and PsycINFO, from their inception to 18 April 2023 (date of search).

#### 2.1.3. Stage 3: Study Selection

After records were uploaded to the Covidence review management software (Veritas Health Innovation Ltd., Melbourne, Australia), deduplicated records underwent title, abstract, and full-text screening. Two reviewers independently assessed each record, with disagreements resolved by a third reviewer. Reviewers met biweekly to discuss study selection and ensure screening consistency.

Studies were deemed relevant for RQ1 if they (1) were original empirical research articles; (2) interventions that included vaccine communication alone or combined with other components (e.g., nutrition); (3) used narrative communication that actively engaged the target audience (e.g., interactivity); (4) delivered narratives via digital health devices (e.g., mobile phones, tablets, etc.) and modalities (e.g., SMS, apps, games for health, etc.), including hybrid approaches combining digital and non-digital elements. For example, in a hybrid approach, a provider might show a vaccination video on a tablet followed by an in-person discussion. Studies were included for RQ2 if they assessed vaccine uptake or intention, and for RQ3 if they examined implementation factors or outcomes (e.g., feasibility, acceptability, etc.).

Articles were excluded if they were (1) not classified as original research articles (e.g., gray literature, etc.); (2) original research articles for which the full text was unavailable; (3) studies published in languages other than English without certified translations from the source. For RQ2 and RQ3, studies reporting only intermediate outcomes (e.g., knowledge, attitudes, beliefs, etc.) without data on vaccination intention or uptake were excluded. For RQ3, studies without empirical data on implementation factors or outcomes were also excluded.

#### 2.1.4. Stage 4: Charting the Data

A data extraction spreadsheet was developed in Microsoft Excel, with a template provided in Supplementary File S2. The data extraction spreadsheet included the following variables: author, title, publication year, study design and period, country, target population, vaccine type(s), purpose or process of vaccine communication, behavioral construct(s) targeted, narrative and classification of narrative, digital health device or modality, and interactive elements of the intervention. One reviewer initially extracted data from each eligible article, followed by a secondary review conducted by another author.

#### 2.1.5. Stage 5: Collating, Summarizing, and Reporting Results

Extracted data from eligible studies were summarized descriptively by RQ. Microsoft Excel was used to organize and compare study characteristics, while Tableau was employed to generate visualizations illustrating key patterns across studies. Results are presented for each study and in aggregate to provide an overall synthesis of the evidence.

Narratives consistent with the study definition were further categorized using Schank and Berman’s classification [18], including: official stories (an innocuous account of events or group positions), invented stories (fictional), firsthand experiential stories (individual experiences), secondhand stories (retellings of others’ experiences), and culturally common stories (pervasive within a cultural context) [18]. Modalities enabling interactivity within interventions were extracted from eligible studies. Digital health interventions were further categorized using the World Health Organization (WHO) classification for digital health, which accounts for device type, delivery modality, and target users [19].

## 3. Results

**Figure 1:**
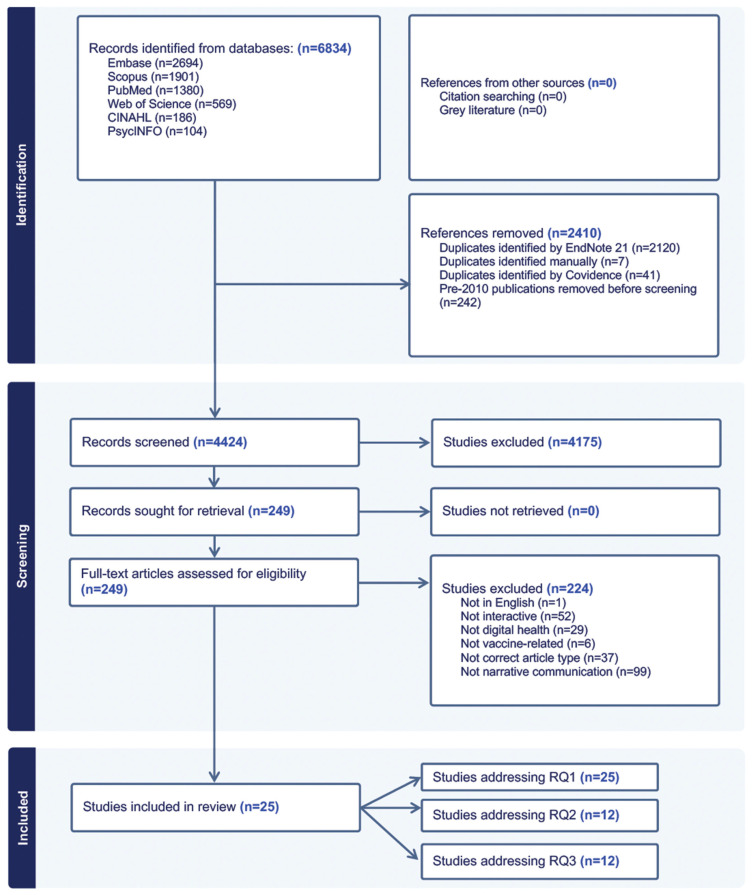
PRISMA flow diagram indicating records identified during the review process and reasons for exclusion. **RQ:** Research Question.

A total of 6834 records were identified and imported into Covidence review management software. After removing 2410 duplicates, 4424 records were retained for title and abstract screening. Of these, 4175 were excluded because they did not address vaccine communication, narratives, interactivity, or digital health. The remaining 249 records underwent a full-text review, resulting in an additional 224 exclusions based on the same criteria. One record was excluded because it was not published in English [20]. Ultimately, 25 studies [21,22,23,24,25,26,27,28,29,30,31,32,33,34,35,36,37,38,39,40,41,42,43,44,45] met the inclusion criteria and addressed RQ1, of which 12 studies [21,23,25,26,31,32,35,38,39,40,42,43] also addressed RQ2, and 12 studies [22,25,26,28,33,34,38,39,40,42,43,44] addressed RQ3 (Figure 1).

### 3.1. RQ1: How Have Interactive, Narrative-Based Digital Health Interventions Been Used for Vaccine Communication?

#### 3.1.1. Characteristics of Included Studies

**Figure 2:**
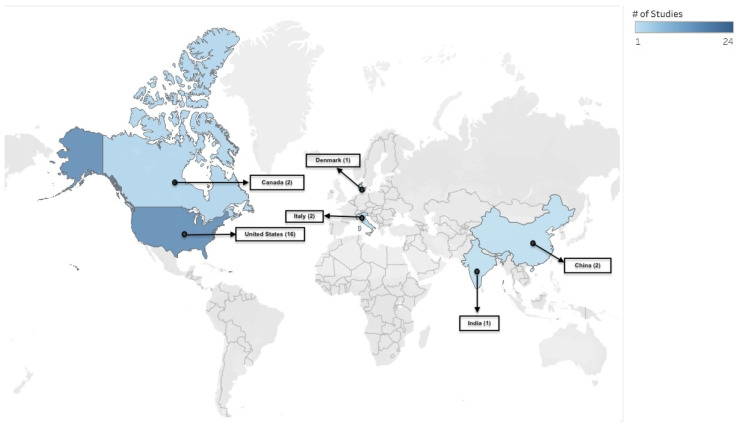
Heat map showing the location of eligible studies. The map is provided for illustrative purposes only and does not imply the expression of any opinion by the authors or their affiliated entities.

Figure 2 presents a heatmap of study locations by country among the 25 eligible studies. The majority of studies were conducted in North America (n = 18) [21,22,23,24,25,26,27,28,29,30,31,32,33,34,35,36,37,38], followed by Europe (n = 3) [39,40,41], and Asia (n = 3) [42,43,44]. One study did not specify its location [45].

Most studies were published in 2020 or later (2020—7 studies [26,28,32,36,41,44,45], 2021—4 studies [22,23,31,42], 2022—8 studies [21,24,29,33,35,37,40,43]), compared to before 2020 (2019—1 study [34], 2018—2 studies [25,39], 2015—1 study [38], 2014—1 study [30], and 2013—1 study [27]). Interventions within studies were categorized using an adapted version of the WHO framework for digital health interventions [14]. Table 1 summarizes the types of digital devices, primary digital modalities, and target users. Most studies (n = 10) were trials [22,26,27,31,34,35,39,42,44,45], and the remainder employed diverse study designs (Table 2).

#### 3.1.2. Study Demographics, Communication Strategies and Objectives

The communication objectives of interventions varied but were broadly grouped into four domains: improving knowledge (n = 15 studies, [22,26,27,28,30,31,34,36,38,39,40,41,42,44,45]), supporting vaccine decision-making (n = 5 studies, [21,24,25,26,33]), shaping perceptions of vaccines (n = 4 studies, [32,36,40,45]), or addressing vaccine misinformation (n = 5 studies, [23,29,35,37,43]). Participants included racially and ethnically varied groups (Black, Somali, Hispanic, Chinese, Korean, South Asian), college students, and parent–child dyads of preteens and adolescents.

#### 3.1.3. Vaccines and Vaccination Communication Purpose

**Figure 3:**
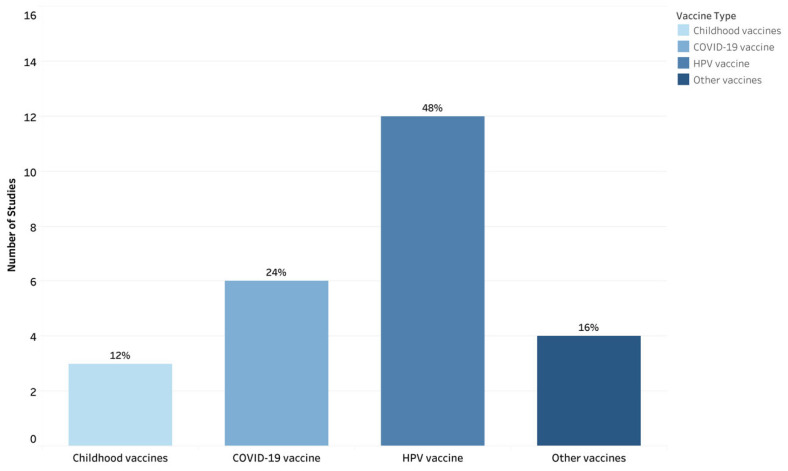
Distribution of Vaccine Types in Eligible Studies (N = 25). Categories are not mutually exclusive, as some studies include multiple vaccines.

Figure 3 represents the distribution of vaccines across eligible studies. Nearly half (n = 12) of the studies focused on the Human Papillomavirus (HPV) vaccine [22,25,26,28,31,33,34,38,40,41,42,45], with two studies having a secondary focus on adolescent vaccines [28] and the influenza vaccine [31]. COVID-19 vaccines were a focus in (n = 6) studies [21,24,29,35,37,43]. There were (n = 3) studies focused on childhood vaccinations [23,30,44]. The remaining (n = 4) studies focused on a range of antigens, including the hepatitis B vaccine [27], influenza vaccine [32], a multi-antigen study that included measles, pertussis, and influenza vaccines [36], and the measles, mumps and rubella (MMR) vaccine [39].

#### 3.1.4. Digital Health Interventions

Interventions that were compatible with more than one device were the most common among (n = 7) studies, delivered through apps, chatbots, and digital storytelling modalities. Mobile-only interventions accounted for (n = 4) of the studies, using apps or web-based interactive technology. Computer-based devices (n = 2) and virtual-reality devices (n = 2) were less frequent. In (n = 10) studies, the compatible device type was not specified, although interventions were delivered through videos, social media, mass media, games, and a photonovel.

#### 3.1.5. Narrative Communication

Table 2 summarizes interactive, narrative-based digital health interventions for vaccine communication. Eight studies incorporated invented narratives. Among these, two studies employed metaphor and symbolic language (e.g., protecting seedlings [the body] with a potion [vaccine]) to frame HPV vaccination as a safeguard for adolescent health [25,26]. One study presented a narrative of a fictional character navigating HPV diagnosis [42], while another used a narrative crafted from the perspectives of parents, adolescents, and clinicians to reflect common HPV vaccine beliefs and to promote vaccination uptake, using accessible, middle school–level language [28]. Two studies focused on COVID-19 vaccine hesitancy: one depicted fictional narrative scenarios shaped by social influences, misinformation, and vaccine fears common among Black young adults [21], and the other used weakened anti-vaccine tropes in short narrative videos to simulate and build resistance to misinformation among unvaccinated individuals [35]. Another study utilized visual simulations to illustrate herd immunity and reinforce community protection [36], while another delivered an immersive VR experience in which participants followed a fictional character in the narrative who unknowingly spread influenza to emphasize the significance of community protection [32].

Six studies employed firsthand experiential narratives. One used a prototype interactive narrative game in which users played the role of doctors interacting with patients modeled on real-life HPV experiences [22]. Another study, which adapted short videos from the National Cancer Institute’s HPV vaccine program, followed narratives of young adults in conversations with peers, health professionals, and parents [33]. Narratives from health professionals and women with cervical cancer were framed as emotionally driven “heart stories” alongside data-oriented “mind stories” to emphasize HPV vaccine safety and effectiveness [41]. A participatory approach invited individuals to create and share digital stories reflecting their own experiences with COVID-19 vaccination [24]. Other strategies included social media posts by influencers sharing their vaccination stories to address COVID-19 vaccine misinformation [29], as well as an app integrated with social media that featured family dialogues on vaccination decisions and experiences with VPDs [30].

Six studies applied culturally tailored narratives, which clustered into three themes. First, community co-design was used to ensure cultural resonance: one study partnered with Somali refugees to create a linguistically tailored MMR narrative that addressed autism-related concerns with input from parents, community leaders, and health workers [23], while another engaged South Asian youth to produce a video narrative on COVID-19 vaccine concerns, including religious objections to ingredients [37]. Second, culturally familiar media formats were applied: a photonovel addressed hepatitis B vaccination concerns among Chinese, Korean, and Vietnamese Americans [27], and a webnovela depicted three young Latinos navigating HPV-related risks, vaccination decision-making, and advocacy within their community [38]. Third, personalized narratives were employed: a study featured Korean American college women of different immigration generations sharing HPV vaccination experiences [34], and another delivered entertaining audio capsules (edutainment) tailored to diverse social groups to communicate the benefits of childhood vaccination [44].

Three studies utilized a secondhand narrative. A study used a narrative from an actress portraying a mother who researched the MMR vaccine and decided to vaccinate her child [39]. Another combined didactic posts (e.g., HPV-related facts) with narrative posts recounting stories of individuals who died from cervical cancer, targeting mothers as key decision makers for HPV vaccination [31]. A third study employed a blog-style narrative written from the perspective of a fictional blogger, depicting either gain-framed experiences of HPV vaccination preventing cancer or loss-framed accounts of forgoing vaccination and later developing cancer [45].

Two studies described the use of official narratives. One study framed HPV vaccination as a normative health behavior, situating it within a broader narrative of everyday self-care practices, including healthy eating, exercise, and hygiene. The presence of a healthcare professional as a central figure in both the animated video and the accompanying game reinforced institutional authority and positioned medical expertise as a trusted guide for vaccination decisions [40]. Similarly, the second study incorporated the WHO’s “3Cs (Confidence, Convenience, and Complacency)” model of vaccine hesitancy into a chatbot intervention that delivered narratives aimed at increasing COVID-19 vaccine acceptance among unvaccinated or booster-hesitant young adults in Hong Kong [43].

#### 3.1.6. Interactivity

Several interventions incorporated interactivity through apps and game-based formats, requiring participants to engage actively with vaccine-related scenarios. A choose-your-own-adventure (CYOA) app prompted users to make decisions within COVID-19 vaccine scenarios shaped by social influences and misinformation [21]. Game-based interventions provided players to role-play as physicians, using action components to target HPV-related cancer cells [22], protect a digital garden with vaccine “potions” [25,26], or sustain engagement through points, rewards, and leaderboards that promoted competition [39,40]. In another approach, an mHealth app required users to respond to digital prompts and voice reminders, supplemented by face-to-face meetings that reinforced positive vaccination behaviors [44].

Interactivity was also supported through immersive and digital platforms that required active user participation. In VR interventions, participants followed a family through their vaccine learning and decision-making process while responding to interactive prompts for self-reflection [23] and navigated scenarios by embodying avatars reduced to the cellular level to explore influenza transmission and immune response [32]. A web application allowed users to create avatars representing themselves and their social networks, which were then embedded into visualizations of herd immunity to illustrate how MMR vaccination influences disease spread and confers protection [36]. Similarly, a web-based interactive technology allowed participants to guide characters’ decisions at key points that shaped narrative trajectories and explored HPV vaccination or consequences of refusal [42]. Chatbots and conversational agents further engaged users by requiring menu-based selections and natural language interactions, including small talk and tailored responses to common questions about COVID-19 vaccination [43].

Interactivity was embedded in mass media and social media strategies [29,31,33,41,45]. A mass media campaign (El Beacon) promoted COVID-19 vaccination by encouraging audience participation through comments and reactions [29], while Facebook-based campaigns on HPV vaccination allowed users to engage with posts through emoji reactions and comments [31], or through interactive calls-to-action that prompted likes, shares, and discussion [41]. Platforms such as Instagram and TikTok similarly fostered real-time engagement through reactions, content sharing, and comments [33,45].

Participatory approaches went further by inviting individuals to co-create digital stories through interactive icebreakers and collectively developing a narrative with each participant contributing a sentence to the narrative about COVID-19 vaccination [24]. Another study used peer-paired storytelling for HPV vaccination, with two participants responding to prompts and building on each other’s reflections [34]. In a youth ambassador program, discussions with families and communities were used to stimulate dialogue and reflection about vaccination [37].

Sequential delivery mechanisms, such as weekly push notifications of narrative episodes, helped sustain engagement across the two-dose HPV vaccine series [28]. Other interventions used digital platforms to facilitate communication, with apps enabling parents to connect with both peers and healthcare providers [30]. A photonovel incorporated interactive “fact boxes” and Q&A sessions to address concerns about the Hepatitis B vaccine [27], while a webnovela used dialogue, thought bubbles, and voiceovers to allow users to explore characters’ experiences with vaccination access and screening [38]. Short inoculation videos incorporated interactivity by requiring participants to answer questions and reflect on content [35].

### 3.2. RQ2: How Have Interactive, Narrative-Based Digital Health Interventions Been Evaluated for Promoting Vaccination Intention or Uptake?

Among the 25 eligible studies, nearly half (n = 12) of the studies assessed vaccination intention (i.e., individuals’ stated willingness or plans to get vaccinated). Intention was measured alone in (n = 8) studies [23,25,32,35,38,39,40,42] and in combination with vaccine uptake (i.e., whether individuals received the vaccine following exposure to the intervention) in (n = 4) studies [21,26,31,43]. Half of the studies (n = 6) focused on the HPV vaccine [25,26,31,38,40,42], and (n = 3) focused on COVID-19 vaccines [21,35,43]. Additionally, three studies solely examined childhood, influenza, and MMR vaccines [23,32,39]. Supplementary File S3 provides details of interventions, evaluation methods, and key findings. Evaluation methods included pre- and post-surveys and qualitative assessments, with sample sizes ranging from 25 to 1953 participants.

Most studies reporting on vaccination intention reported positive effects, particularly for HPV, MMR, and COVID-19 vaccines, whereas the impact on the influenza vaccine was limited. Macario et al. [38] observed a 65.5% increase in vaccination intention after exposure to a culturally tailored webnovela. Occa et al. [40] reported a mean score increase on a 5-point Likert scale from 3.20 to 3.80 in an animated video condition and from 2.93 to 3.73 in the game-based intervention. Wang et al. [42] found that within the narrative condition, interactivity reduced information avoidance and increased the intention to receive HPV vaccination among young women (M = 5.51, SD = 1.47) compared with non-interactive conditions (M = 4.72, SD = 1.46). Cates et al. [25] reported that preteens were motivated to receive HPV vaccination after playing a serious game, while parents expressed more cautious support in qualitative FGDs. Streuli et al. [23] found that in a VR prototype intervention, 54% of parents felt more comfortable with MMR vaccination, and 83% indicated they would recommend it to others after exposure to the intervention. Piltch-Loeb et al. [35] found that participants who viewed inoculation videos were more likely to recognize misinformation, less willing to share misinformation and disinformation, and more willing to vaccinate compared with controls. Fadda et al. [37] similarly reported increased MMR vaccination intention following a gamified mobile-phone intervention. By contrast, Nowak et al. [32] found that interventions using VR, video, and electronic pamphlets had a limited impact on influenza vaccination intention.

Among studies reporting both vaccination intention and uptake, all showed positive effects across both outcomes. For the COVID-19 vaccine, Stoner et al. [21] reported that 75% (n = 112) were fully vaccinated, 7% (n = 10) had received one dose, and 19% (n = 28) were unvaccinated; 47% (n = 71) of the fully vaccinated intended to receive a booster. Luk et al. [43] found an increase in vaccination intention among unvaccinated participants (from 3.0 to 3.9; *p* < 0.001) and booster-hesitant participants (from 1.9 to 2.8; *p* < 0.001), with uptake at 4 months in 18 (82%) of 22 and 7 (29%) of 24 participants, respectively. For the HPV vaccine, Cates et al. [26] reported higher initiation (22% vs. 15%) and completion (9% vs. 2%) rates in the intervention group compared with the control group. Buller et al. [31] found initiation increased from 63.4% at baseline to 71.3% at 12 months and 73.3% at 18 months (*p* < 0.001), while uptake of two or more doses increased from 50.2% to 62.5% and 65.9% over the same period (*p* < 0.001).

### 3.3. RQ3: What Implementation Factors Are Associated with the Use of Narrative-Based Digital Health Interventions for Vaccine Communication?

A detailed summary of methods and key findings related to implementation factors and outcomes is provided in Supplementary File S4. Of the 25 eligible studies, nearly half (n = 12) reported on implementation factors and outcomes. Acceptability was the most frequently assessed (n = 6) [26,28,34,38,42,43], followed by usability (n = 5) [22,25,28,39,43] and feasibility (n = 4) [26,38,40,44]. One study each assessed usefulness [39], adaptation [33], and adoption [44]. The HPV vaccine was the most common (n = 9) studies [22,25,26,28,33,34,38,40,42], followed by the MMR [39], COVID-19 [43], and childhood vaccines [44]. Interventions ranged from serious games, mobile and web-based apps, chatbots, videos, web-based interactive tools, and social media strategies. Evaluation methods ranged from mixed methods approaches to quantitative surveys (e.g., System Usability Scale, pre- and post-intervention surveys) and qualitative FGDs.

In studies reporting on acceptability, participants responded positively to culturally appropriate interventions. For example, in a pilot RCT, Korean college women who viewed a tailored narrative video reported more favorable attitudes toward HPV vaccination than those who received written information [34]. Similarly, a webnovela designed for Latinas and health professionals was described as entertaining, motivational, and age-appropriate; however, some users did not engage with the interactive elements due to limited navigational cues [37]. In contrast, a study among young Chinese college students found that web-based content presented via data visualizations was not readily accepted, leading to increased information avoidance; however, when interactivity was embedded within the narrative format, it significantly reduced information avoidance [42].

Usability outcomes across interventions were generally favorable. A prototype interactive serious game focused on HPV vaccination achieved a mean System Usability Scale score of 73.6, surpassing the standard usability benchmark of 68 [22]. In another serious game, preteens and parents valued the game’s entertaining and educational features, such as earning tokens, progressing through levels, and completing knowledge-based challenges related to HPV [25]. Similarly, in an app designed to promote HPV vaccine uptake, adolescents and parents expressed intent to use the app within two weeks, citing the relevance of its core features, including a vaccine tracker, videos, stories, and a forum [28].

Feasibility was demonstrated through high engagement and implementation potential across various delivery formats. For example, an intervention combining mHealth components (e.g., educational audio capsules and voice-based immunization reminders) with face-to-face community mobilization activities was found to be highly feasible. All pre-specified criteria for recruitment, randomization, retention, and contamination were successfully met, and uptake was high across both channels, including among families, suggesting strong potential for scalability in real-world settings [44]. Likewise, a study using FGDs and an embedded experiment with children showed that both an evidence-based animated video and a web-based game about the HPV vaccine were well received and improved children’s knowledge and perceptions. No single message format emerged as more effective than the other [40].

In a study evaluating the usefulness of a mobile application, participants described MorbiQuiz, which was designed to increase parents’ knowledge of the MMR vaccine, as highly educational and valuable for supporting informed vaccination decision-making and encouraging further information seeking among parents and prospective parents [39]. Similarly, a study that adapted the National Cancer Institute’s video-based HPV Vaccine Decision Narratives program for dissemination on Instagram, TikTok, and Twitter using the Push–Pull and RE-AIM frameworks found that all platforms increased following, with Instagram and TikTok outperforming Twitter across impressions, engagement, and reach metrics. While TikTok achieved the greatest reach (unique accounts viewing content), Instagram generated the highest gains in followers, engagement, and impressions [33]. In Tika Vaani (“vaccine voice” in Hindi), a community-based intervention delivered through small face-to-face meetings, adoption (e.g., uptake of the intervention) was nearly universal (50% ex-ante vs. 94% in practice), demonstrating strong interest and acceptability [44].

### 3.4. Protocol Deviations

Two planned analyses were not undertaken. For RQ1, we did not assess the outcomes of narratives (e.g., congruence with personal values, memorability, perceived realism) as these outcomes were rarely reported and described too inconsistently to permit meaningful synthesis. For RQ3, we did not examine barriers and facilitators to implementation, as the included studies were primarily focused on reporting implementation outcomes.

## 4. Discussion

### 4.1. Principal Findings

This scoping review identified 25 studies describing interactive, narrative-based digital health interventions for vaccine communication (RQ1). Among those, twelve studies assessed vaccination outcomes (intention or uptake, RQ2). Twelve studies examined implementation factors (RQ3), with acceptability being the most frequently reported outcome (n = 6 studies). The purpose of communication varied in the studies, but they were broadly aimed at improving vaccine knowledge, supporting decision-making, shaping perceptions, or addressing misinformation. Delivery formats included devices (e.g., phones, computers, tablets), computer-based platforms, and virtual reality. Interactivity was achieved through gamification (e.g., leaderboards, rewards), immersive virtual reality, and social media functions (e.g., likes, comments). Nearly half of the studies (n = 12) focused on HPV vaccination, reflecting interest in adolescent and young adult vaccination, suggesting that interactive, narrative-based digital health interventions are most likely to be acceptable and feasible among this demographic. In contrast, few studies examined childhood vaccines or adult vaccines (e.g., influenza), highlighting a critical gap in the literature. With vaccine communication needs continuing to evolve in the aftermath of the COVID-19 pandemic, extending these interventions to a broader range of antigens and population groups should be a priority for future research.

Notably, most interventions were designed primarily for a target demographic (e.g., young adults, parents, etc.), with limited attention to healthcare providers. For example, a study incorporated click-to-call reminders within a smartphone app [30], and another assessed providers’ perceptions of a culturally tailored webnovela [38]. Given evidence that provider recommendations influence vaccine uptake [46,47], eligible studies lacked assessment of the acceptability and feasibility of integrating interactive, narrative-based digital health interventions into clinical settings and provider workflows. Further investigation is needed to determine how and which types of such interventions can be embedded within healthcare settings to optimize vaccine uptake.

In addition, (n = 10) studies did not specify the device type used, reporting only the digital modality. This lack of detail hinders understanding of how delivery channels impact accessibility and engagement, making it challenging to determine whether interventions reach underserved groups (e.g., by age, gender, or location) or risk perpetuating digital divides. Furthermore, all eligible studies in this scoping review were conducted in high- and middle-income countries, which limits the applicability of the findings to low-resource settings where digital access and health system contexts differ.

Related to narratives, no studies directly compared different narrative types, limiting insight into which formats are most engaging for different audiences. No studies examined the mediating processes of narratives (e.g., identification, transportation, etc.) and their effects on vaccination intention or uptake. Moreover, while most studies drew on behavioral theories [21,22,25,26,28,30,31,33,35,36,38,40,42], only one drew exclusively on narrative communication theory [34]. Future studies may benefit from drawing on narrative-specific theories to inform intervention design and provide theoretical grounding, thereby clarifying how narrative features contribute to behavior change.

While interventions in eligible studies offered opportunities for interactivity, the depth of interactivity was generally rudimentary. For example, interactivity was often limited to simple navigation features or, in the case of mass media and social media strategies [29,31,33,41,45], to basic functions such as emojis, comments, and shares. To determine whether interactivity meaningfully enhances engagement with such interventions, future research should move beyond describing their interface features to measuring how users interact with them and whether this engagement supports vaccine uptake. One framework that may be useful in this regard is proposed by Cole-Lewis et al. [48], which distinguishes between “Little e” engagement (e.g., with the digital behavior change intervention) and “Big E” engagement (e.g., with the targeted health behavior). Applying such distinctions during intervention design may help clarify how different levels of user engagement contribute to behavior change. Qualitative and co-design studies may help identify which interactive features, informed by behavioral theory, are perceived as credible and motivating, followed by mixed-methods and pilot studies to assess these measures in relation to vaccination knowledge, attitudes, and behaviors. Insights from formative research could then inform factorial designs to determine which forms of interactivity most effectively resonate with and influence vaccination behaviors across broader populations.

There were also a few comparisons between narrative and non-narrative interventions and vaccination outcomes. Evidence on mechanisms was also limited, with little examination of whether interactivity increased the effects of interventions or shaped vaccination-related behaviors. Future research should compare interactive and non-interactive formats to clarify the contribution of interactivity to vaccine communication interventions, while leveraging behavioral theory to develop more nuanced understandings of what theoretically grounded interactivity entails and how it supports vaccination-related behavior change.

Among the eligible studies, only 12 studies examined vaccination intention (n = 8) or both intention and uptake (n = 4). Culturally tailored interventions improved vaccination intention, suggesting that cultural relevance may increase motivation towards vaccination. However, variation in how intention and uptake were operationalized limited comparability across studies. Structural and contextual factors (e.g., access, cost, and provider recommendation) that mediate the gap between intention and uptake were not consistently identified across studies. In addition, study designs varied widely in their evaluation methods and sample sizes, with inconsistent definitions and measures of intention and uptake, which complicated cross-study comparisons. Future research should address these gaps by incorporating validated, longitudinal measures of both intention and uptake, expanding to a broader range of vaccines, and employing rigorous designs that allow for clearer attribution of vaccination outcomes.

In the 12 studies that reported on implementation, the most frequently reported factors or outcomes were acceptability and usability, either exclusively or in combination [22,25,26,28,34,38,39,42,43], with positive responses noted particularly for culturally tailored and entertaining interventions [34,38]. Most studies (n = 9) that reported on implementation focused on HPV vaccines [22,25,26,28,33,34,38,40,42], and future research should expand to include other vaccines. Moreover, future research should extend implementation research to other implementation factors and outcomes, such as adoption, adaptation, and long-term sustainability.

### 4.2. Strengths and Limitations

This scoping review has several methodological strengths. First, to our knowledge, this is the first review to focus specifically on the intersection of narratives and interactivity via digital modalities in vaccine communication, providing a distinct contribution to the field. Second, we conducted comprehensive data extraction across multiple domains, including narrative types, interactive features, implementation outcomes, and behavioral measures. Third, the inclusion of both qualitative and quantitative studies provided a more nuanced understanding of the intervention’s impact, while the focus on implementation added practical relevance. Lastly, the review encompassed diverse populations and vaccine contexts, including a wide range of delivery formats such as games, apps, videos, VR, and social media, thereby increasing its breadth and applicability to inform future research and practice.

Several limitations should be taken into consideration. First, only studies published in the English language were included, which may have excluded relevant studies published in other languages. Second, the search was limited to selected academic databases, and gray literature was excluded, which raises the possibility of missing relevant articles. Third, all included studies were conducted in high and middle-income settings, which may limit the generalizability of the findings to low-income countries. Fourth, while some studies described a comprehensive structure or the development of the narrative, others lacked sufficient details of how narratives were constructed. In such cases, narrative elements were identified based on how closely they aligned with the definition. Finally, although data on vaccination intention and uptake, and implementation factors were extracted, the review did not include a formal risk of bias assessment for each study, which limits the ability to fully evaluate the quality of the included evidence.

## 5. Conclusions

This scoping review of 25 studies provides a comprehensive synthesis of interactive, narrative-based digital health interventions for vaccine communication. Findings highlight the diversity of narrative formats, interactive features, and digital health platforms and modalities used to engage various populations and address vaccines and research gaps. These interventions show promise in influencing vaccination attitudes, intentions, and, to a lesser extent, uptake. While many studies reported positive user perceptions and implementation potential, few incorporated long-term follow-up, standardized outcome measures, or robust assessments of vaccine uptake. Future research should prioritize rigorous, longitudinal designs; expand focus to include a broader range of vaccines, populations, and countries; and explore how interactivity and narrative elements can be optimized to support sustained vaccine confidence and decision-making.

## Figures and Tables

**Table 1:** Characteristics of Digital Health Interventions among eligible studies (N = 25 studies).

Device Type & No. of Studies (N=)	Primary Digital Modality	Target User
Multi-device compatibility (e.g., mobile, computer, or tablet) (N = 7) [21,24,25,26,28,38,43]	App [21,25,26,28]; Chatbot [43]; Webnovela [38]; Digital Health Intervention with Digital Storytelling [24]	Parent-adolescent dyads [25,26,28]; Latinas and Health professionals [38]; young Black or African American adults [21,24]; young adults [43]
Mobile (N = 4) [30,39,42,44]	App [30,39,44]; Web-based Interactive Technology [42]	Parents [30,39,44]; Chinese Female Undergraduates [42]
Computer (N = 2) [36,40]	Web Application [36]; Animated Video and Web-based Game [40]	Men and Women [36]; Middle School Children [40]
Virtual Reality (N = 2) [23,32]	360° Video Animation [23]; VR, video, or e-pamphlet [32]	Somali Adults [23]; Vaccine-avoidant Adults [32]
Not Specified (N = 10) [22,27,29,31,33,34,35,37,41,45]	Social Media [31,33,41]; Blog on social media [45]; Mass media [29]; Videos [35,37]; Storytelling video [34]; Prototype Interactive Narrative Game [22]; Photonovel [27]	College students [22,45]; Hispanic communities [29]; Asian Americans [27]; Korean American women [34]; South Asian Youth [37]; Youth and Parents [33]; Mothers of Daughters [31,41]; Unvaccinated Individuals [35]

App: Application, VR: Virtual reality.

**Table 2:** Characteristics of studies included in the scoping review on interactive, narrative-based digital health interventions for vaccine communication (N = 25 studies).

Author, Year, & Country	Study Design & Demographics (N=)	Vaccine	Theoretical Underpinnings or Behavioral Constructs	Digital Health Intervention, & Communication Objective or Process	Interactive Narrative
Stoner, et al. (2022), USA [21]	Online survey; Young Black or African American adults (N = 150)	COVID-19 vaccine	Social cognitive theory	CYOA (Tough Talks) App communicated COVID-19 vaccination by addressing vaccine, provider, and system mistrust.	Narratives simulated vaccine decision-making scenarios shaped by social influences, fears, and misinformation. Interactivity involved selecting responses to each scenario.
Zhu et al. (2021), USA [22]	RCT; College students (N = 99)	HPV vaccine	Self-determination theory	A serious game (Vaccination Vacation) communicated the HPV virus and vaccine by integrating real-life experiences to improve vaccine awareness.	Real-life patient narratives inspired the game’s characters. Interactivity was facilitated via an action component using a virtual machine to attack cells of HPV-related cancers. The game challenged players to convince characters to receive vaccination by selecting responses from a pool of options.
Streuli, et al. (2021), USA [23]	Community-based participatory research; Somali adults (N = 60 refugees, N = 7 expert advisors)	Childhood vaccines	Information retention and behavior change	The VR addressed vaccine misinformation through a culturally and linguistically tailored approach.	Narrative followed an expectant mother who discovered the MMR vaccine at a doctor’s visit, explored MMR vaccine science, and debunked of MMR-autism myth. Interactive prompts within the VR encouraged user self-reflection.
Maragh-Bass, et al. (2022), USA [24]	Feasibility study & demonstration; Young Black Adults (N = 11)	COVID-19 vaccine	Decision-making	Culturally tailored digital health intervention (Tough Talks: COVID) empowered young Black people to make autonomous decisions about COVID-19 vaccine receipt.	Narrative addressed vulnerability to COVID-19, vaccine hesitancy, countered misinformation, and community connections. Digital storytelling workshops were interactive and featured icebreakers, with participants co-creating a narrative and each contributing a sentence.
Cates et al. (2018), USA [25]	Focus group discussions; Pre-teens (N = 16), and mothers (N = 9)	HPV vaccine	Self-determination theory and health belief model	Serious game (Land of Secret Gardens) educated preteens about HPV infection and vaccine while ensuring the content was age-appropriate and captured their interest.	An educational narrative on HPV infection and vaccination. Interactive mini games, such as finding hidden objects and defending plants from the HPV virus. Completing tasks earned rewards, allowing players to “protect” their garden (body) with a vaccine-symbolizing “potion”.
Cates et al. (2020), USA [26]	Pilot randomized controlled trial; Parent-preteen dyads (N = 55)	HPV vaccine	Self-determination theory	Serious game (Land of Secret Gardens) educated preteens about HPV infection and vaccination and encouraged dialogue with parents and healthcare providers. For healthcare providers, communication materials comprised brochures, posters, and interactive online training.	Same as Cates et al. [25].
Lee, et al. (2013), USA [27]	Randomized community trial; Chinese, Korean, and Vietnamese (N = 441)	Hepatitis B vaccine	Self-efficacy and intention	Culturally tailored (photonovel) was developed to increase awareness of Hepatitis B vaccination and screening via PowerPoint presentation, role-play video, and a Q&A session.	The narrative depicted storylines from common life experiences in the community and key concerns about Hepatitis B. Actors, photography, and a respected physician played as actors for cultural relevance. Interactive “fact boxes” and a Q&A section increased engagement and provided additional information.
Teitelman, et al. (2020), USA [28]	Development and usability study; Adolescents (N = 20; aged 11–14 years) and parents (N = 34)	Primary: (HPV vaccine); secondary: (adolescent vaccines)	Integrated behavioral model	An app (Vaccipack) provided vaccine information and addressed parental beliefs influencing adolescent’s HPV vaccination decisions.	Narratives comprised 26 short stories written from the perspectives of parents, adolescents, and clinicians. The app was interactive and delivered a new story weekly via push notifications, promoting sustained engagement over the six-month period required for the two-dose HPV vaccine series.
Silesky, et al. (2022), USA [29]	Media monitoring study; Hispanic adults	COVID-19 vaccine	Attitudes and beliefs	A (mass media communication strategy) including radio PSAs, op-eds, satellite media tours, website, and social media platforms like Instagram and Facebook via (El Beacon) was developed to counter COVID-19 vaccine misinformation and promote uptake through fact-based messaging delivered by trusted community voices.	The narrative featured prompts debunking common COVID-19 vaccine misinformation and sharing positive, personal vaccination messages tailored to the Hispanic community. Prompts were regularly updated to reflect evolving misinformation, and an interactive format encouraged engagement through comments and likes.
Peck, et al. (2014), USA [30]	Descriptive study design; Parents or guardians (N = 262, average age 34–35 years)	Childhood vaccines	Health belief model	A Call the Shots (CTS) app served as a vaccination reminder system for parents and provided information on vaccines recommended at each provider visit.	Narrative featured personal stories from families affected by VPDs. The CTS platform was interactive, allowing parents to connect with other parents through social media, as well as their child’s provider.
Buller, et al. (2021), USA [31]	RCT; Mothers (N = 869, mean age of 43.1 years), with N = 469 teenage daughters (mean age of 15.3 years)	HPV vaccine; secondary: (influenza vaccine)	Social cognitive, transportation, and diffusion of innovations theories	A social media campaign (Health Chat) was delivered in 2 Facebook groups to promote vaccination. Posts incorporated social norms-based appeals, appearance-based and health-risk messaging.	Some of the posts in the social media campaign were in narrative format (e.g., a story about someone who died from cervical cancer). The interactive feature included reactions to posts (likes, loves, sad) and comments.
Nowak, et al. (2020), USA [32]	One-way between-subjects experimental design; Adults (N = 171, age of 18–49 years)	Influenza vaccine	Perceptions, beliefs, confidence, and intentions	Whether the use of (VR, video, or e-pamphlet) could improve influenza-related perceptions, beliefs, confidence, and intentions among individuals who avoid vaccination.	Narrative depicted an individual spreading the flu in public, while the interactive VR component shrank the individual to a cellular level, immersing participants in a storyline about flu transmission, immune response, and vaccine benefits.
Hopfer, et al. (2022), USA [33]	Observational study; Youth and young adults (11–26 years), and parents of adolescents (N = NS)	HPV vaccine	Centering and discourse coherence theories	A social media strategy, via Instagram, TikTok, and Twitter, was utilized to distribute videos that presented vaccine decision narratives told by youth through informal conversation.	Thirteen short videos featured narratives set in diverse social contexts (e.g., peer, doctor, and parent conversations) emphasizing the benefits of HPV vaccination. Interactivity was encouraged through commenting, emoji reactions, and content sharing.
Kim, et al. (2019), USA [34]	RCT; Korean or Korean American female students (N = 104, mean age of 21.7 years)	HPV vaccine	Storytelling/Narrative communication theory	An HPV video intervention promoted HPV vaccination through culturally relevant storytelling.	An interactive peer-paired storytelling approach, with two storytellers engaging in a dialogue, responding to interviewer prompts, and building on each other’s reflections.
Piltch-Loeb, et al. (2022), USA [35]	Quasi-experimental Trial; Unvaccinated individuals (N = 1991, mean age of 40.7 years)	COVID-19 vaccine	Inoculation theory	30-s inoculation videos aimed to promote resistance against persuasion against COVID-19 vaccine misinformation.	The narrative was presented in video formats (fact-based, rhetoric-focused, and hybrid), which countered anti-vaccine misinformation through an inoculation video warning of manipulation techniques and a stimulus video demonstrating them. Interactive component asked participants to answer questions evaluating their perceptions of the content.
Hakim, et al. (2020), Canada [36]	Development study; Men and women (N = 110, mean age of 38 years)	Measles, pertussis, and influenza vaccines	Health belief model, Gestalt visual principles, cognitive theory of multimedia learning, and affect heuristic	A (web application) communicated the concept of community immunity to individuals with diverse educational backgrounds while simultaneously assessing their cognitive and emotional responses.	The narrative illustrated community immunity, while the interactive feature allowed participants to create avatars of themselves and their social circles. These avatars were incorporated into a two-minute visualization showing how vaccination coverage and social interactions affect disease spread.
Kandasamy, et al. (2022), Canada [37]	Cross-sectional and one-group pretest–post-test design; South Asian youth (N = 30, aged 18–29 years)	COVID-19 vaccine	Knowledge and confidence	A digital media health program (SAY-VAC) consisted of a video and an information sheet to address COVID-19 vaccine concerns, and improve confidence.	A culturally tailored narrative promoted COVID-19 vaccination and addressed concerns (e.g., vaccine ingredients). Interactivity was facilitated by youth Ambassadors who used the video to initiate discussions with family and community members and encourage vaccination.
Macario, et al. (2015), USA [38]	Formative research (pre- and post-survey, focus group discussion, online survey); Latinas (N = 84), and health professionals (N = 41)	HPV vaccine	Social cognitive theory, exchange theory, the health belief model, the theory of reasoned action, the trans-theoretical model of health behavior change, and diffusion of innovations	A (webnovela) addressed knowledge gaps about higher HPV incidence and cervical cancer among Latinas. The webnovela incorporated voice-overs, still photos, short video clips, and the use of culturally relevant language (e.g., Spanglish).	The narrative followed three young Latino friends who discovered HPV and its consequences, tracing their reactions, information-seeking, and decisions to vaccinate and promote vaccination. The interactive features included dialog, thought bubbles, and voiceovers, which allowed users to explore the characters’ journeys through vaccination access and screening.
Fadda, et al. (2018), Italy [39]	Randomized control trial; Women and men (N = 140)	MMR vaccine	Knowledge, confidence, decision-making, and psychological empowerment	Two mobile-based apps (MorbiQuiz) aimed to improve parents’ MMR vaccine knowledge and decision-making: one used gamified questions, the other employed video narratives and messages to increase psychological empowerment.	The narrative featured an actress playing a mother who recounted how she became empowered to vaccinate her child with MMR. The interactive component included a gamified intervention that awarded points (stars) for correct answers, with a leaderboard enabled for score comparison and competition.
Occa, et al. (2022), Italy [40]	Mixed Methods Study; Middle school students (N = 35, aged 11–12 years)	HPV vaccine	Theory of planned behavior and social cognitive theory	An animated video and web-based game (Salut e HPV or Health and HPV) were utilized as part of a communication strategy to increase children’s knowledge and positive perceptions about HPV and HPV vaccination.	The narrative featured a healthcare professional who addressed questions and concerns, normalized HPV vaccination, and supported character identification. The interactive game allowed children to choose characters, track progress, earn points, receive feedback while promoting engagement, autonomy, and skill development.
Pedersen, et al. (2020), Denmark [41]	Evaluation of social media campaign; (Primary target: mothers of daughters aged 10–14 years; secondary target: fathers)	HPV vaccine	Attitudes	A social media strategy, including Facebook, Instagram, and YouTube, communicated messages about the safety and effectiveness of the HPV vaccine.	Personal cervical cancer narratives and health professional experiences were featured to increase authenticity and relevance. Interactive calls-to-action encouraged viewer engagement through likes, shares, and comments.
Wang, et al. (2021), China [42]	RCT; Chinese (N = 180 female undergraduate students)	HPV vaccine	Theory of information avoidance and the limited capacity model of motivated mediated messages	A (web-based interactive technology) sought to influence young females’ intention to vaccinate by allowing them to read or interact with content.	The narrative followed XiaoA, a young woman diagnosed with HPV, through four phases of her experience: diagnosis, work life, relationships, and a medical consultation. An interactive condition allowed participants to guide her decisions at key points, shaping her journey and exploring potential consequences of different decisions.
Luk, et al. (2022), China [43]	Pre-post pilot study; Chinese adults (N = 290, mean age of 21.4)	COVID-19 vaccine	Knowledge and decision-making	Chatbot (Vac Chat, Fact Check) communicated information about COVID-19 disease and vaccination.	The narrative was delivered via a chatbot in a conversational format with predefined rules and natural language processing. The chatbot covered information on COVID-19 disease, vaccination benefits, myths, boosters, and service access. Interactive menu options were utilized for user engagement.
Johri, et al. (2020), India [44]	Cluster-randomized pilot Trial; Households (N = 387 with children aged 0–12 months)	Childhood vaccines	Knowledge and attitudes	mHealth mobile app (Tika Vaani) educated beneficiaries about vaccination and dispelled vaccine-related misinformation.	The narrative was delivered through entertainment educational audio capsules promoting vaccination and child health. The interactive component included voice reminders and face-to-face meetings to educate and engage beneficiaries about vaccination and child health.
Lee, et al. (2020), NS [45]	Randomized online experiment; College students (N = 220, mean age of 22.74)	HPV vaccine	Perceived social norms, perceived similarity, framing (gain vs. loss), and personal experience	A (social media personal blog) was developed to investigate whether a personal narrative can influence perceived social norm, specifically examining the role of perceived similarity with the blogger and social media metrics in shaping perceptions.	Four stimulus narratives featured either gain-framed (HPV vaccination prevents cancer) or loss-framed (cancer risk from non-vaccination). An interactive manipulation of reads, shares, and comments was used to assess perceptions, engagement, and perceived credibility or relatability of the narratives.

CYOA: Choose-your-own-adventure, MMR: Measles-mumps-rubella, NS: Not specified, RCT: Randomized control trial, USA: United States, VPDs: Vaccine-preventable Diseases.

## Supplementary Materials

The following supporting information can be downloaded at: https://www.mdpi.com/article/10.3390/vaccines13121220/s1, File S1: Search Strategy; File S2: Data Extraction Template; File S3: Evaluation of Interventions; File S4: Implementation Factors.

## Abbreviations

App	Application
CYOA	Choose-your-own-adventure
HPV	Human Papillomavirus
MMR	Measles, mumps, and rubella
NS	Not specified
PRISMA-ScR	Preferred Reporting Items for Systematic reviews and Meta-Analyses extension for Scoping Reviews
RCT	Randomized control trial
RQ	Research Question
USA	United States
VPDs	Vaccine-preventable Diseases
VR	Virtual Reality
WHO	World Health Organization

## References

[1] Shattock A.J., Johnson H.C., Sim S.Y., Carter A., Lambach P., Hutubessy R.C.W., Thompson K.M., Badizadegan K., Lambert B., Ferrari M.J. (2024). Contribution of vaccination to improved survival and health: Modelling 50 years of the Expanded Programme on Immunization. Lancet.

[2] Larson H.J., Schulz W.S., Tucker J.D., Smith D.M. (2015). Measuring vaccine confidence: Introducing a global vaccine confidence index. PLoS Curr..

[3] Borges do Nascimento I.J., Pizarro A.B., Almeida J.M., Azzopardi-Muscat N., Gonçalves M.A., Björklund M., Novillo-Ortiz D. (2022). Infodemics and health misinformation: A systematic review of reviews. Bull. World Health Organ..

[4] MacDonald N.E. (2015). Vaccine hesitancy: Definition, scope and determinants. Vaccine.

[5] Xu J. (2023). A Meta-Analysis Comparing the Effectiveness of Narrative vs. Statistical Evidence: Health vs. Non-Health Contexts. Health Commun..

[6] Zebregs S., van den Putte B., Neijens P., de Graaf A. (2015). The differential impact of statistical and narrative evidence on beliefs, attitude, and intention: A meta-analysis. Health Commun..

[7] Braddock K., Dillard J.P. (2016). Meta-analytic evidence for the persuasive effect of narratives on beliefs, attitudes, intentions, and behaviors. Commun. Monogr..

[8] Shelby A., Ernst K. (2013). Story and science: How providers and parents can utilize storytelling to combat anti-vaccine misinformation. Hum. Vaccines Immunother..

[9] Dube E., Trottier M.E., Greyson D., MacDonald N.E., Meyer S.B., MacDonald S.E., Driedger S.M., Witteman H.O., Ouakki M., Gagnon D. (2024). Use of narratives to enhance childhood vaccine acceptance: Results of an online experiment among Canadian parents. Hum. Vaccines Immunother..

[10] Conlin J., Kumble S., Baker M., Shen F. (2024). Re-Routing Persuasion: How Conversion Messages Boost Attitudes and Reduce Resistance Among Holdouts Unvaccinated for COVID-19. Health Commun..

[11] Hinyard L.J., Kreuter M.W. (2007). Using narrative communication as a tool for health behavior change: A conceptual, theoretical, and empirical overview. Health Educ. Behav..

[12] Winskell K., Sabben G., Obong’o C. (2019). Interactive Narrative in a Mobile Health Behavioral Intervention (Tumaini): Theoretical Grounding and Structure of a Smartphone Game to Prevent HIV Among Young Africans. JMIR Serious Games.

[13] Haji Said A., Winskell K., Bednarczyk R.A., Reardon E.E., Vasudevan L. (2024). Interactive Narrative-Based Digital Health Interventions for Vaccine Communication: Protocol for a Scoping Review. JMIR Res. Protoc..

[14] World Health Organization (2019). Recommendations on Digital Interventions for Health System Strengthening. https://www.who.int/publications/i/item/9789241550505.

[15] Lo Moro G., Ferrara M., Langiano E., Accortanzo D., Cappelletti T., De Angelis A., Esposito M., Prinzivalli A., Sannella A., Sbaragli S. (2023). Countering vaccine hesitancy: A systematic review of interventions to strengthen healthcare professionals’ action. Eur. J. Public Health.

[16] Arksey H., O’Malley L. (2005). Scoping studies: Towards a methodological framework. Int. J. Soc. Res. Methodol..

[17] Tricco A.C., Lillie E., Zarin W., O’Brien K.K., Colquhoun H., Levac D., Moher D., Peters M.D.J., Horsley T., Weeks L. (2018). PRISMA Extension for Scoping Reviews (PRISMA-ScR): Checklist and Explanation. Ann. Intern. Med..

[18] Schank R.C., Berman T.R., Green M.C., Strange J.J., Brock T.C. (2002). The pervasive role of stories in knowledge and action. Narrative Impact: Social and Cognitive Foundations.

[19] World Health Organization (2018). Classification of Digital Health Interventions V1.0. https://www.who.int/publications/i/item/WHO-RHR-18.06?.

[20] Galhardi C., Freire N., Marques Fagundes M., Minayo M., Cunha I. (2022). Fake news e hesitação vacinal no contexto da pandemia da COVID-19 no Brasil. Ciênc. Saúde Coletiva.

[21] Stoner M.C.D., Tweedy D., Comello M.G.L., Toval C., Pettifor A.E., Larsen M.A., Baez A., Maragh-Bass A.C., Tolley E.E., Browne E.N. (2022). Using narratives to inform the development of a digital health intervention related to COVID-19 vaccination in Black young adults in Georgia, North Carolina and Alabama. Vaccine.

[22] Zhu A., Amith M., Tang L., Cunningham R., Xu A., Boom J.A., Tao C. (2021). Experimenting with a Prototype Interactive Narrative Game to Improve Knowledge and Beliefs for the HPV Vaccine. HCI Int Late Break. Pap..

[23] Streuli S., Ibrahim N., Mohamed A., Sharma M., Esmailian M., Sezan I., Farrell C., Sawyer M., Meyer D., El-Maleh K. (2021). Development of a culturally and linguistically sensitive virtual reality educational platform to improve vaccine acceptance within a refugee population: The SHIFA community engagement-public health innovation programme. BMJ Open.

[24] Maragh-Bass A., Comello M.L., Tolley E.E., Stevens D., Wilson J., Toval C., Budhwani H., Hightow-Weidman L. (2022). Digital Storytelling Methods to Empower Young Black Adults in COVID-19 Vaccination Decision-Making: Feasibility Study and Demonstration. JMIR Form. Res..

[25] Cates J.R., Fuemmeler B.F., Diehl S.J., Stockton L.L., Porter J., Ihekweazu C., Gurbani A.S., Coyne-Beasley T. (2018). Developing a Serious Videogame for Preteens to Motivate HPV Vaccination Decision Making: Land of Secret Gardens. Games Health J..

[26] Cates J.R., Fuemmeler B.F., Stockton L.L., Diehl S.J., Crandell J.L., Coyne-Beasley T. (2020). Evaluation of a Serious Video Game to Facilitate Conversations About Human Papillomavirus Vaccination for Preteens: Pilot Randomized Controlled Trial. JMIR Serious Games.

[27] Lee S., Yoon H., Chen L., Juon H.S. (2013). Culturally appropriate photonovel development and process evaluation for hepatitis B prevention in Chinese, Korean, and Vietnamese American communities. Health Educ. Behav..

[28] Teitelman A.M., Gregory E.F., Jayasinghe J., Wermers Z., Koo J.H., Morone J.F., Leri D.C., Davis A., Feemster K.A. (2020). Vaccipack, A Mobile App to Promote Human Papillomavirus Vaccine Uptake Among Adolescents Aged 11 to 14 Years: Development and Usability Study. JMIR Nurs..

[29] Silesky M.D., Panchal D., Fields M., Peña A.S., Diez M., Magdaleno A., Frausto-Rodriguez P., Bonnevie E. (2023). A Multifaceted Campaign to Combat COVID-19 Misinformation in the Hispanic Community. J. Community Health.

[30] Peck J.L., Stanton M., Reynolds G.E. (2014). Smartphone preventive health care: Parental use of an immunization reminder system. J. Pediatr. Health Care.

[31] Buller D.B., Pagoto S., Henry K., Berteletti J., Walkosz B.J., Bibeau J., Baker K., Hillhouse J., Arroyo K.M. (2021). Human Papillomavirus Vaccination and Social Media: Results in a Trial with Mothers of Daughters Aged 14–17. Front. Digit. Health.

[32] Nowak G.J., Evans N.J., Wojdynski B.W., Ahn S.J.G., Len-Rios M.E., Carera K., Hale S., McFalls D. (2020). Using immersive virtual reality to improve the beliefs and intentions of influenza vaccine avoidant 18-to-49-year-olds: Considerations, effects, and lessons learned. Vaccine.

[33] Hopfer S., Phillips K.K., Weinzierl M., Vasquez H.E., Alkhatib S., Harabagiu S.M. (2022). Adaptation and Dissemination of a National Cancer Institute HPV Vaccine Evidence-Based Cancer Control Program to the Social Media Messaging Environment. Front. Digit. Health.

[34] Kim M., Lee H., Kiang P., Allison J. (2019). Development and acceptability of a peer-paired, cross-cultural and cross-generational storytelling HPV intervention for Korean American college women. Health Educ. Res..

[35] Piltch-Loeb R., Su M., Hughes B., Testa M., Goldberg B., Braddock K., Miller-Idriss C., Maturo V., Savoia E. (2022). Testing the Efficacy of Attitudinal Inoculation Videos to Enhance COVID-19 Vaccine Acceptance: Quasi-Experimental Intervention Trial. JMIR Public Health Surveill..

[36] Hakim H., Bettinger J.A., Chambers C.T., Driedger S.M., Dubé E., Gavaruzzi T., Giguere A.M.C., Kavanagh É., Leask J., MacDonald S.E. (2020). A Web Application About Herd Immunity Using Personalized Avatars: Development Study. J. Med. Internet Res..

[37] Kandasamy S., Ariyarajah A., Limbachia J., An D., Lopez L., Manoharan B., Pacht E., Silver A., Uddandam A., Vansjalia K.M. (2022). South Asian Youth as Vaccine Agents of Change (SAY-VAC): Evaluation of a public health programme to mobilise and empower South Asian youth to foster COVID-19 vaccine-related evidence-based dialogue in the Greater Toronto and Hamilton Area, Canada. BMJ Open.

[38] Macario E., Matiella A.C. (2015). A bilingual webnovela on the human papillomavirus: Will Latinas and health professionals use it?. J. Commun. Healthc..

[39] Fadda M., Galimberti E., Fiordelli M., Schulz P.J. (2018). Evaluation of a Mobile Phone-Based Intervention to Increase Parents’ Knowledge About the Measles-Mumps-Rubella Vaccination and Their Psychological Empowerment: Mixed-Method Approach. JMIR mHealth uHealth.

[40] Occa A., Stahl H.M., Julien-Bell S. (2022). Helping Children to Participate in Human Papillomavirus-Related Discussions: Mixed Methods Study of Multimedia Messages. JMIR Form. Res..

[41] Pedersen E.A., Loft L.H., Jacobsen S.U., Søborg B., Bigaard J. (2020). Strategic health communication on social media: Insights from a Danish social media campaign to address HPV vaccination hesitancy. Vaccine.

[42] Wang Q., Zhang W. (2021). The use of Web-based interactive technology to promote HPV vaccine uptake among young females: A randomized controlled trial. BMC Womens Health.

[43] Luk T.T., Lui J.H.T., Wang M.P. (2022). Efficacy, Usability, and Acceptability of a Chatbot for Promoting COVID-19 Vaccination in Unvaccinated or Booster-Hesitant Young Adults: Pre-Post Pilot Study. J. Med. Internet Res..

[44] Johri M., Chandra D., Kone K.G., Sylvestre M.P., Mathur A.K., Harper S., Nandi A. (2020). Social and Behavior Change Communication Interventions Delivered Face-to-Face and by a Mobile Phone to Strengthen Vaccination Uptake and Improve Child Health in Rural India: Randomized Pilot Study. JMIR mHealth uHealth.

[45] Lee T.K., Su L.Y. (2020). When a Personal HPV Story on a Blog Influences Perceived Social Norms: The Roles of Personal Experience, Framing, Perceived Similarity, and Social Media Metrics. Health Commun..

[46] Lin C., Mullen J., Smith D., Kotarba M., Kaplan S.J., Tu P. (2021). Healthcare Providers’ Vaccine Perceptions, Hesitancy, and Recommendation to Patients: A Systematic Review. Vaccines.

[47] de Koning R., Gonzalez Utrilla M., Spanaus E., Moore M., Lomazzi M. (2024). Strategies used to improve vaccine uptake among healthcare providers: A systematic review. Vaccine X.

[48] Cole-Lewis H., Ezeanochie N., Turgiss J. (2019). Understanding Health Behavior Technology Engagement: Pathway to Measuring Digital Behavior Change Interventions. JMIR Form. Res..

