# Supplementary material for: Interactive, Narrative-Based Digital Health Interventions for Vaccine Communication: Scoping Review
Source: Vaccines (Basel). 2025 Dec 2;13(12):1220. doi: 10.3390/vaccines13121220 (PMC12737697; doi:10.3390/vaccines13121220)
Supplement: Supplementary file 1 [file vaccines-13-01220-s001.zip › S1-Search Strategy.pdf]

Search string used for identifying relevant records in PubMed

((“Narration”[Mesh] or Communication[Mesh]) or (narrat*[tw] or storytell*[tw] or story-tell*[tw] or “story telling”[tw] or storyline*[tw] or story[tw] or stories[tw] or conversation*[tw] or testimoni*[tw])) AND ((“Social Media”[Mesh] or “Mobile Applications”[Mesh] or “Smartphone”[Mesh] or “Telemedicine”[Mesh] or “Artificial Intelligence”[Mesh] or “Digital Technology”[Mesh] or “Computers”[Mesh]) or (digital[tw] or app[tw] or apps[tw] or “social media”[tw] or twitter[tw] or facebook[tw] or tiktok[tw] or instagram[tw] or Weibo[tw] or youtube[tw] or telegram[tw] or Whatsapp[tw] or Tumblr[tw] or Pinterest[tw] or snapchat[tw] or wechat[tw] or reddit[tw] or myspace[tw] or computer*[tw] or smartphone*[tw] or chat[tw] or blog*[tw] or game[tw] or gaming[tw] or games[tw] or gamification[tw] or weblog*[tw] or online[tw] or web-based[tw] or electronic[tw] or ehealth[tw] or e-health[tw] or “electronic health”[tw] or mhealth[tw] or m-health[tw] or “mobile health”[tw] or “artificial intelligence”[tw] or ai[tw] or “machine learning”[tw] or “deep learning”[tw] or chatbot[tw] or “chat bot”[tw] or chatgpt[tw])) AND ((“Vaccines”[Mesh] or “Vaccination”[Mesh]) or (vaccin*[tw] or immuniz*[tw] or immunis*[tw] or anti-vax*[tw] or antivax*[tw] or “anti vax”[tw] or anti-vaccin*[tw])) AND (English[Filter]
