# Supplementary material for: Interactive, Narrative-Based Digital Health Interventions for Vaccine Communication: Scoping Review
Source: Vaccines (Basel). 2025 Dec 2;13(12):1220. doi: 10.3390/vaccines13121220 (PMC12737697; doi:10.3390/vaccines13121220)
Supplement: Supplementary file 1 [file vaccines-13-01220-s001.zip › S2-Data Extraction Template.pdf]

Data Extraction Template for Individual Studies

Field	Description or Example
Author	First author of the research article
Title of Research Article	Full title of study
Year of Publication	Year the study was published
Study Duration	Length of the study
Study Design (N=)	Methodological design (e.g., randomized controlled trial, qualitative, etc.) and the total number of participants included
Country	Location where the study was conducted.
Population Type and Age	Specific group of individuals and age range of participants
Vaccine Type(s)	Vaccine(s) addressed in the study (e.g., HPV, Hepatitis B, etc.)
Vaccine Communication Purpose	Stated aim of the communication (e.g., to improve vaccine uptake, address vaccine hesitancy, increase knowledge, correct misinformation, etc.)
Communication Process	(e.g., sender, channel, message, receiver, and feedback)
Behavioral Constructs Targeted	Psychological or behavioral determinants the intervention aimed to influence (e.g., self-efficacy, perceived risk, social norms, etc.)
Narrative Theory (if applicable)	Theoretical framework underpinning the use of narratives (e.g., transportation theory, inoculation theory, entertainment–education)
Content of Narrative	Specific storyline or themes conveyed in the narrative (e.g., personal experiences, fictional accounts, etc.)
Schank and Berman's classification of narrative	Classification of the narrative type (official, invented, first-hand experiential, second-hand, and culturally common)
Interactivity	Form of user engagement integrated into the intervention (e.g., reflection prompts, push notifications, etc.).
Digital Health Device	Hardware through which the intervention was accessed (e.g., smartphone, tablet, etc.)
Digital Health Modality	Medium or channel of delivery (app, video, etc.)
Vaccination Intention and/or uptake	Reported outcomes related to participants' willingness to vaccinate or actual vaccination
Implementation Outcomes	How the intervention functioned in practice (e.g., feasibility, acceptability, etc.)
Barriers and facilitators to implementation	Factors influencing implementation success or challenges
