# Supplementary material for: Interactive, Narrative-Based Digital Health Interventions for Vaccine Communication: Scoping Review
Source: Vaccines (Basel). 2025 Dec 2;13(12):1220. doi: 10.3390/vaccines13121220 (PMC12737697; doi:10.3390/vaccines13121220)
Supplement: Supplementary file 1 [file vaccines-13-01220-s001.zip › S3-Evaluation of Interventions.pdf]

Evaluation of interactive, narrative-based digital health interventions associated vaccination intention or uptake (n=12 studies)						
**Author**	**Intervention**	**Study Design**	**Evaluation Method(s) & Demographics**	**Vaccine**	**Vaccination Intention or Uptake**	**Key Findings**
Stoner, et al. [20]	CYOA (Tough Talks) app	Online survey	Survey (N=150)	COVID-19 vaccine	VI/VU	• Survey results showed that 75% (n=112) of respondents were fully vaccinated, 7% (n=10) had received one dose and planned to complete the series, and 19% (n=28) were unvaccinated.<br>• Among the unvaccinated, 9% (n=14) preferred to wait and see how the vaccine was working for others.<br>• Of those fully vaccinated, 47% (n=71) intended to receive a booster shot, reported before the CDC's recommendation for all adults aged 18 and older.
Streuli, et al. [22]	VR platform	Community-based participatory research	5 FGDs (N=57)<br>Survey (N=17)	Childhood vaccines	VI	• Findings from FGDs showed that 13 (54%) of 24 participants reported feeling more comfortable or much more comfortable with vaccination than they were prior to the exposure to the prototype intervention.<br>• Findings from FGDs showed that 20 (83%) of 24 participants stated they would recommend MMR vaccination to others in their community.
Cates, et al. [24]	Serious game (Land of Secret Gardens)	Focus group discussions	3 FGDs (N=16 preteens) and (N=9 parents)	HPV vaccine	VI	• Preteens expressed a desire to get vaccinated to protect themselves and stay healthy.<br>• Parents were generally supportive of HPV vaccination, but also noted that some remained hesitant and wanted more time to decide.
Cates, et al. [25]	Serious game (Land of Secret Gardens)	Pilot randomized controlled trial	Pre- and post-survey (N=55 dyads completed baseline surveys and N=47 dyads completed post survey)<br><br>3 FGDs (N=7 preteens)	HPV vaccine	VI/VU	• Vaccine initiation was higher in the intervention group compared to the control group (22% vs. 15%), as were completion rates (9% vs. 2%).<br>• Only (1 of 27) in the control group completed the HPV vaccine series, whereas (5 of 28) participants in the intervention group completed the vaccine series.<br>• Most participants who initiated vaccination in the intervention group were still within the recommended timeframe for completing the HPV vaccine series.
Macario, et al. [37]	Webnovela	Formative research	Pre- and post-survey (N=84)	HPV vaccine	VI	• Vaccination intention increased by 65.5% post-intervention (among 29 to 48 participants).

Buller, et al. [30]	Social media campaign (Health Chat)	Randomized Control Trial	Pre- and post-survey (N=869 mothers and N=469 daughters)	HPV vaccine	VI/VU	• HPV vaccine initiation (1 dose) was reported by 63.4% of mothers at baseline, increasing to 71.3% at 12 months and 73.3% at 18 months post-intervention (p<.001).<br>• HPV vaccine uptake (2–3 doses) increased from 50.2% at baseline to 62.5% at 12 months and 65.9% at 18 months post-intervention (p<.001).
Nowak, et al. [31]	VR, video, or e-pamphlet	One-way between-subjects experimental design	Pre- and post-survey (N=171)	Influenza vaccine	VI	• Flu vaccination intention was low across all conditions, with a mean score of 2.15 on a 5-point scale.
Piltch-Loeb, et al. [34]	Inoculation videos	Quasi-experimental Trial	Pre- and post intervention questionnaire (N=1,953 all groups)	COVID-19 vaccine	VI	• The intervention group had the second-smallest P value across the three study outcomes (F(3,1929)=4.1, P=.01). This result remained significant after Holm adjustment, where the threshold was .05/2 = .03. Least-squares means for the control and the three intervention arms were 2.77 (0.09), 3.05 (0.09), 3.05 (0.09), and 3.05 (0.09), respectively. Compared with the control group, scores in the Narrative, Fact, and Hybrid conditions were each 0.28 points higher (P= .012, .011, and .010), indicating greater willingness to receive the COVID-19 vaccine among participants compared to controls.
Fadda, et al. [38]	App (MorbiQuiz)	Randomized control trial	Post-test questionnaire (N=140)<br><br>Qualitative interviews (N=60)	MMR vaccine	VI	• Participants who received the knowledge intervention had a significantly higher intention to vaccinate (t179=2.111; P=.03) and greater confidence in their vaccination decision (t179=2.76; P=.006) compared to the control group.<br>• Findings from interviews found that parents would benefit from clear guidance and comparative viewpoints on vaccinations, as excessive pressure without factual support can cause frustration and emotional distress. Educational efforts alone may not greatly boost vaccination acceptance.
Occa, et al. [39]	An animated video and web-based game (Salut e HPV or Health and HPV)	Mixed Methods Study	9 FGDs (N=35)<br><br>Pre- and post-intervention questionnaire (N=35)	HPV vaccine	VI	• In the animated video condition, vaccination intention increased from a mean of 3.20 (SD=1.19) pre-intervention to 3.80 (SD=0.83) post-intervention.<br>• In the game condition, vaccination intention increased from 2.93 (SD=1.10) to 3.73 (SD=0.70) post-intervention.

Wang, et al. [41]	Web-based interactive technology	Randomized controlled trial	Survey (N=180)	HPV vaccine	VI	• A post-hoc analysis showed that under the narrative condition, interactive content (M=5.51, SD=1.47) led to higher intention to receive the HPV vaccine compared to non-interactive content (M=4.72, SD=1.46).<br>• In the data visualization condition, non-interactive content (M=5.21, SD=1.71) resulted in higher vaccination intention than interactive content (M=4.63, SD=1.81).
Luk, et al. [42]	Chatbot, (Vac Chat, Fact Check)	Pre-post pilot study	Pre- and post-evaluation (N=46)	COVID-19 vaccine	VI/VU	• Among unvaccinated participants, the intention to vaccinate increased from 3.0 to 3.9 (P<.001).<br>• Among booster-hesitant participants, intention to receive a booster increased from 1.9 to 2.8 (P<.001).<br>• At a post hoc 4-month follow-up, 18 (82%) out of 22 of initially unvaccinated participants and 7 (29%) out of 24 of booster-hesitant participants reported receiving a COVID-19 vaccine or booster, respectively.

[a] VI: Vaccination Intention

[b] VU: Vaccine uptake

[c] NS: Not specified

[d] FGDs: Focus Group Discussions
