# Supplementary material for: Interactive, Narrative-Based Digital Health Interventions for Vaccine Communication: Scoping Review
Source: Vaccines (Basel). 2025 Dec 2;13(12):1220. doi: 10.3390/vaccines13121220 (PMC12737697; doi:10.3390/vaccines13121220)
Supplement: Supplementary file 1 [file vaccines-13-01220-s001.zip › S4-Implementation Factors.pdf]

Implementation factors associated with interactive, narrative-based digital health interventions for vaccine communication (n=12 studies)						
**Author**	**Intervention**	**Vaccine**	**Implementation Factor(s)**	**Study Design**	**Evaluation Method(s)**	**Key Findings**
Zhu et al. [21]	Serious game (Vaccination Vacation)	HPV vaccine	Usability	Randomized control trial	SUS (N=99)	• System usability score for the game was 73.6, which exceeds the average score of 68 reported in previous studies, though the difference was not statistically significant (t(43) =1.80, p=0.08). According to this study, prior studies classify this score as “good,” indicating acceptable usability.
Cates, et al. [24]	Serious game (Land of Secret Gardens)	HPV vaccine	Usability	Focus group discussions	FGD (N=7)	• Preteens and parents appreciated the game’s entertaining and educational elements, including earning tokens, advancing through levels, and the garden-based metaphor.<br>• Preteens found the music and embedded HPV-related facts particularly engaging. Parents supported the incorporation of knowledge-based levels to reinforce learning.<br>• Some parents expressed hesitation about using games as motivational tools and questioned their real-world impact.
Cates, et al. [25]	Serious game (Land of Secret Gardens)	HPV vaccine	Acceptability and feasibility	Pilot randomized controlled trial	Physical/Emotional/Narrative Presence Scale (N=18)<br><br>3 FGDs (N=7 preteens)	• More than half of participants reported positive perceptions of game autonomy, competence, ease of use, and freedom during gameplay.<br>• At the same time, more than half of participants described the game as boring, emotionally unengaging, and unable to sustain their attention.
Fadda, et al. [38]	App (MorbiQuiz)	MMR vaccine	Usability and usefulness	Randomized control trial	Web-based survey (N=140)<br><br>Qualitative interviews (N=60)	• Participants who received the knowledge intervention (quiz), either alone or combined with the empowerment intervention (videos/messages), reported significantly more favorable perceptions of the app compared to those in the empowerment-only group ($F_{2,137}$=15.335; P<.001).

						• Parents in the empowerment-only group expressed dissatisfaction, citing a lack of substantive information to support autonomous decision-making about MMR vaccination.<br>• Qualitative interviews highlighted parents viewed the quiz-based intervention as an active, engaging learning experience, in contrast to the videos, which were perceived as passive.<br>• The knowledge intervention was valued for its gamified elements that promoted information acquisition, challenged existing beliefs, and encouraged information-seeking behavior.
Luk, et al. [42]	Chatbot (Vac Chat, Fact Check)	COVID-19 vaccine	Usability and acceptability	Pre-post pilot study	SUS (N=46)	• The median SUS score was 72.5 (IQR 65–77.5) out of 100, indicating good usability.<br>• On a 5-point scale, median (IQR) perceived usefulness scores were:<br>- **4** for getting information about the COVID-19 vaccine.<br>- **3** for making vaccination decisions.<br>- **3** for increasing motivation to get vaccinated.<br>- The median recommendation score was **7** out of 10, suggesting moderate overall acceptability.
Macario, et al. [37]	Webnovela	HPV vaccine	Acceptability and feasibility	Formative research	10 FGDs (N=84)	• Participants expressed the webnovela was interactive, entertaining, educational, amusing, colorful, easy to read, and motivational.<br>• Most participants felt the communication format was appealing and appropriate for women in their age group, and some considered it potentially useful for school-aged children.<br>• Most participants were unaware that tabs within the webnovela could be clicked to access additional information or alternate endings, due to the absence of clear visual cues.

Wang, et al. [41]	Web-based interactive technology	HPV vaccine	Acceptability	Randomized controlled trial	Survey (N=180)	• Findings suggested that web-based interactive technology may be acceptable, however, narratives may improve understanding, acceptability, and effectiveness of the technology.<br>• In the narrative condition, interactivity (compared to non-interactivity) decreased information avoidance and increased intention to receive the HPV vaccine.<br>• In contrast, within the data visualization condition, interactivity had no significant effect on vaccination intention.
Teitelman, et al. [27]	App (Vaccipack)	Primary (HPV vaccine), secondary (adolescent vaccines)	Usability and acceptability	Development and usability study	Survey (N=54)	• Acceptability was high across both groups: 88% of parents and 75% of adolescents found the app easy to use, while 82% of parents and 85% of adolescents viewed it as beneficial.<br>• 75% of adolescents and 88% of parents indicated that they intended to use the app within the next two weeks.
Occa, et al. (2022), Italy [39]	An animated video and web-based game (Salut e HPV or Health and HPV)	HPV vaccine	Feasibility	Mixed-methods study	9 FGDs (N=35)<br><br>Embedded Experiment (Pre- and post-intervention questionnaire (N=35)	• Both qualitative and experimental results showed the 2 educational materials were well received and increased children's intention to discuss the HPV vaccine from pre- to post-exposure. Children responded positively to the characters in the animated video and game.<br>• Children suggested making the video and game available on the web and across multiple social media platforms to improve accessibility for themselves and their parents.<br>• Children expressed a desire for more information on possible side effects of the vaccine and symptoms of HPV infection, in addition to the risk of cancer. One criticism involved the appearance of the characters, as some children felt the characters looked too young, which reduced their relatability.

Hopfer, et al. [32]	Social media strategy	HPV vaccine	Adaptation	Observational study	Observational study (N=NS)	• All platforms showed growth in followers, but Instagram and TikTok outperformed Twitter in terms of impressions, engagement, followers, and reach.<br>• TikTok had the highest reach by generating the most views from unique accounts, while Instagram led in follower growth, engagement, and overall impressions.<br>• Among the 12 strategies that were tested on Instagram, 6 contributed to increasing reach.
Johri, et al. [43]	mHealth mobile app (Tika Vaani)	Childhood vaccines	Feasibility and adoption		Households (N= 387)<br><br>Pre- and post-survey	• Adoption of the interventions was high, with 94% (173/184) of participants engaging in at least one new intervention.<br>• Participation rates were 78.3% (144/184) participants for the face-to-face channel and 67.4% (124/184) participants for the mHealth channel. Additionally, 38.0% (70/184) of households reported using the mHealth intervention on a weekly basis.<br>• Findings supported the feasibility of conducting a larger-scale study and highlight the potential for population-level health impact.
Kim, et al. [33]	HPV video intervention	HPV vaccine	Acceptability		Korean or Korean American female undergraduate or graduate students<br><br>Pilot RCT (N=104)	• Participants in the intervention group (n=54), who viewed a culturally tailored storytelling video, reported significantly higher satisfaction than those in the comparison group (n=50), who received written information (P<0.05).<br>• Greater endorsement was observed for videos that aligned with participants' cultural and generational experiences.

[a] FGDs: Focus Group Discussions

[b] SUS: System Usability Survey

[c] NS: Not specified
